# Effects of subclinical mastitis on automatic milking system data, hematological and biochemical parameters, and milk composition in Holstein cows

**DOI:** 10.5713/ab.24.0460

**Published:** 2024-08-27

**Authors:** Mooyoung Jung, Seogjin Kang, Eunjeong Jeon, Dong-Hyun Lim, Donghyeon Kim, Jin San Moon, Sang-Hwan Hyun, Seungmin Ha

**Affiliations:** 1 National Institute of Animal Science, Rural Development Administration, Cheonan 31000, Korea; 2 Bacterial Disease Division, Animal and Plant Quarantine Agency, Gimcheon 39660, Korea; 3 School of Veterinary Biosecurity and Protection, Chungbuk National University, Cheongju, 28644, Korea

**Keywords:** *Bos Taurus*, Mammary Glands, Subclinical Inflammation

## Abstract

**Objective:**

Subclinical mastitis decreases milk production and quality, despite the normal appearance of the mammary glands and milk. Herein, we aimed to investigate changes in factors monitored via automatic milking systems (AMS) prior to subclinical mastitis onset and identify differences in hematological and biochemical parameters and milk composition at subclinical mastitis onset.

**Methods:**

Thirty-two Holstein cows were divided into two groups according to somatic cell counts (SCC) from AMS and milk composition analysis and the California mastitis test (CMT): healthy cows (controls [CON], n = 16, SCC <500×10^3^ cells/mL and negative for CMT) and cows with subclinical mastitis (SCM, n=16, SCC ≥500×10^3^ cells/mL and positive for CMT). Eventually, 121 milk samples from the CON ([mCON], n = 60) and SCM ([mSCM], n = 61) groups were obtained; SCM samples were categorized as those from non-inflamed (mNQ) or subclinically-inflamed (mIQ) quarters. We evaluated AMS factors; hematological, biochemical, and milk composition parameters; and bacterial isolation.

**Results:**

In cows with SCM, milk yield decreased, and electrical conductivity (EC) changed before disease onset. Milk EC decreased in mNQ although increased in mIQ (p<0.05). The SCM group had higher globulin levels and lower basophil counts; albumin-to-globulin ratio; and total cholesterol, albumin, and blood urea nitrogen levels than the CON group (p<0.05). The mIQ group had higher SCC but lower levels of lactose and milk solids-not-fat than those in the mCON and mNQ groups (p<0.05). The mCON group had higher levels of milk non-protein nitrogen than the mNQ group (p<0.05). Opportunistic mastitis pathogens were isolated in the mIQ group.

**Conclusion:**

Changes in milk yield and EC measured using AMS occurred prior to subclinical mastitis, which may be associated with variation in basophil counts; albumin-to-globulin ratio; and total cholesterol, albumin, blood urea nitrogen, globulin, SCC, milk lactose, and milk solids-not-fat levels at disease onset. These findings provide new insights into early-stage subclinical mastitis.

## INTRODUCTION

Mastitis, a ubiquitous disease with high incidence and prevalence worldwide, causes direct and indirect damage to the dairy industry, including decreased milk production and quality, discarded milk, additional costs for diagnostic tests and treatment, culling, death, and animal welfare problems [1]. Mastitis can be categorized into clinical and subclinical forms according to the visible milk condition of the mammary quarters [2,3]. Clinical mastitis, ranging from mild (abnormal milk only) to severe (abnormal milk, abnormal mammary glands, and systemic clinical signs), is characterized by visibly abnormal milk from the mammary quarters. Subclinical mastitis is characterized by normal-appearing mammary glands and visibly normal milk despite decreased milk production, lowered milk quality, and high somatic cell counts (SCC). Subclinical mastitis is approximately 40 times more prevalent than clinical mastitis, owing to the difficulty in its detection [4].

Field-applicable diagnostic methods have been used to detect subclinical mastitis on dairy farms. The California mastitis test (CMT) is conventionally used in farms because it is rapid, economical, and practical [5]. Automatic digital devices have been newly developed for on-farm subclinical mastitis detection [6]. Infrared thermography is effective in detecting quarters with subclinical mastitis [7]. Automatic milking systems (AMS) detect the occurrence of subclinical mastitis at an early stage by measuring SCC, milk electrical conductivity (EC), and lactate dehydrogenase levels in milk [7–9].

Previous studies have demonstrated an association between subclinical mastitis and hematological and serum biochemical parameters in dairy cows [10–13]. Studies have shown that dairy cows with subclinical mastitis have increased neutrophil and aspartate aminotransferase levels and decreased total protein levels [10,11,13]. However, findings regarding serum calcium and phosphorus levels differ [10, 12,13]. In addition, other hematological and serum biochemical parameters have not been investigated in association with subclinical mastitis.

Milk produced by quarters with subclinical mastitis has a composition different from that of milk produced by healthy quarters in cattle. Cows with subclinical mastitis produce milk with decreased lactose, solids-not-fat, calcium, iron, phosphorous, potassium, and zinc levels [10,14–17]. However, previous studies have reported varying results regarding the levels of fat, protein, and total solids in the milk of cows with subclinical mastitis [10,14,15,17].

The aims of this study were to explore changes in factors monitored via automatic health monitoring and AMS before the incidence of subclinical mastitis and to identify differences in hematological and serum biochemical parameters and milk composition between cows with subclinical mastitis and healthy cows. We hypothesized that unusuality monitored via automatic health monitoring and AMS may occur in cows with subclinical mastitis prior to the onset of the disease, and that blood and milk compositions in cows with subclinical mastitis may differ from those in healthy cows.

## MATERIALS AND METHODS

### Ethics approval

This work was approved by the Institutional Animal Care and Use Committee (IACUC) at the National Institute of Animal Science, the Republic of Korea (approved numbers: NIAS-2020127 and NIAS-2022102). All experimental procedures involving animals were conducted in strict accordance with relevant guidelines and regulations. All methods used for the present study were in accordance with REFLECT (Reporting Guidelines for Randomized Controlled Trials for Livestock and Food Safety) guidelines.

### Animals and case definition

This observational study was conducted between February 2022 and May 2023 on a farm of the study’s host institution in Southeast Asia, in order to examine the factors associated with subclinical mastitis by analyzing the data from AMS and analyses of blood and milk samples. In total, 32 Holstein cows were included. The animals were raised in a loose barn with sawdust bedding that housed around 30 dairy cows at a time. They were milked on a Lely Astronaut A4 milking robot (Lely Industries NV, Maassluis, the Netherlands) with free cow traffic and fed total mixed rations comprising concentrates, soybean meal, corn silage, alfalfa hay, timothy hay, enzyme, minerals, and vitamin additives *ad libitum*.

We defined subclinical mastitis using three procedures: the alarms from the AMS, results from the CMT, and SCC obtained from milk composition analyses. The threshold for mastitis used in this study was 500×10^3^ cells/mL since the AMS triggers an alert to indicate a mammary health problem when the system detects milk with 500×10^3^ cells/mL. In addition, the cutoff level of 500×10^3^ cells/mL is utilized when employing the CMT [18]. We examined alerts from the AMS (Lely Astronaut A4 milking robot) at 9 A.M. Once the AMS (Lely Astronaut A4 milking robot) alarmed cows with >500×10^3^ cells/mL, we performed the CMT on milk obtained from a mammary quarter. Among cows of similar age, parity, and days in milk in the same herd, we randomly selected a control cow with <500×10^3^ cells/mL and negative CMT results. Milk samples were immediately analyzed with a milk composition analyzer (Combiscope FTIR 300 HP; Delta Instruments B.V., Drachten, the Netherlands). Cows with <500×10^3^ cells/mL, as measured by the AMS and milk composition analyzer, and negative CMT results were categorized in the control group (CON; n = 16), whereas those with >500×10^3^ cells/mL and positive CMT results were categorized in the subclinical mastitis group (SCM; n = 16).

### Measurement of body temperature and sample collection

On completion of CMT, body temperature was measured in the rectum using a thermometer. Then, blood and milk samples were obtained. Blood was drawn from the jugular vein and collected using ethylenediaminetetraacetic acid and serum-separating tubes from 32 cows (CON and SCM). Milk samples were obtained manually with clean hands using disposable conical tubes from each quarter in the morning. We obtained milk samples from the CON group before obtaining samples from the SCM group. In total, 121 milk samples (milk samples from the control group [mCON], n = 60; milk samples from the SCM group [mSCM], n = 61) were obtained from the groups, as four cows in the CON and three cows in the SCM groups had one non-functional quarter of the udder. In addition, mSCM (n = 61) was categorized as samples from non-inflamed quarters ([mNQ], n = 34, <500×10^3^ cells/mL) and those from subclinically-inflamed quarters ([mIQ], n = 27, >500×10^3^ cells/mL). The samples were immediately sent to a laboratory located on the farm.

### Data collection from automatic systems

We collected data from the automatic health monitoring and AMS from 3 days prior to the incidence of subclinical mastitis. The rumination time and activity were measured using tags attached to the collars (HR-Tag; SCR Engineers Ltd., Netanya, Israel). Milk temperature, yield, and EC were measured using the Lely Astronaut A4 milking robot. Milk temperature and yield were measured for individual cows, whereas EC of the udder quarter was measured separately. Milk yield was aggregated daily. Milk yield per udder quarter was calculated by dividing the milk yield by the number of milk-producing udder quarters.

### Laboratory analyses of blood and milk

The complete blood count (CBC) test and serum biochemical and mineral analyses were performed on 32 dairy cows (CON, n = 16; SCM, n = 16) as previously described [19]. The CBC profile included three types of parameters: erythrocytes (red blood cell count, hematocrit, hemoglobin, mean corpuscular volume, mean corpuscular hemoglobin, mean corpuscular hemoglobin concentration, red cell distribution width, and reticulocyte count); leukocytes (white blood cell, neutrophil, lymphocyte, monocyte, eosinophil, and basophil counts); and platelets (platelet count, mean platelet volume, platelet distribution width, and plateletcrit). The levels of glucose, non-esterified fatty acids, triglyceride, total cholesterol, total protein, albumin, blood urea nitrogen (BUN), creatinine, alanine transaminase, aspartate transaminase, alkaline phosphatase, gamma-glutamyl transferase, lactate dehydrogenase, creatine kinase, calcium, magnesium, and inorganic phosphorus were measured. The globulin level was calculated by subtracting the albumin level from the total protein level. The albumin-to-globulin (A/G) ratio was calculated by dividing the albumin level by the globulin level.

Analysis of 121 milk samples (mCONs, n = 60; mNQ, n = 34; mIQ, n = 27) was conducted to measure the SCC and levels of fat, protein, lactose, citrate, total solids, non-protein nitrogen, and solids-not-fat using a Combiscope FTIR 300 HP.

### Bacteria isolation in milk samples from subclinically inflamed quarters

Bacterial examination for bovine mastitis from the sample with the SCC ≥500×10^3^ cells/mL using a milk composition analyzer (Combiscope FTIR 300 HP; Delta Instruments B.V., the Netherlands) was conducted using standard laboratory techniques. Bacterial isolation in milk samples was performed as previously described [20]. Briefly, 10 μL of milk samples were streaked on 5% blood agar plates (Komed, Seongnam, Korea) and incubated at 37°C for 48 h. After incubation, colonies were identified by matrix-assisted laser desorption ionization–time of flight mass spectrometry (bioMérieux, Marcy l’Etoile, France), according to the manufacturer’s instructions.

### Statistical methods

Statistical analyses were performed using the Statistical Package for the Social Sciences software (version 27.0; IBM Corp., Armonk, NY, USA). The Shapiro–Wilk and Levene tests were used for normality analysis and equality of variances for the independent *t*-test, paired *t*-test, and one-way analysis of variance (ANOVA). The Mann–Whitney U with Bonferroni’s method, Wilcoxon signed-rank test, and Kruskal–Wallis test were used for parameters that did not satisfy the normality analysis or equality of variances. A generalized linear mixed model with the Bonferroni correction was used to evaluate repeated measurements of milk temperature, yield, and EC. In the generalized linear mixed model with the Bonferroni correction, time and group were fixed effects, whereas cows nested within the group were random effects. Multiple comparison analyses among the groups were conducted using the Bonferroni post-hoc test for one-way ANOVA and the Mann–Whitney U test with Bonferroni’s method for Kruskal–Wallis tests. Data are expressed as the mean± standard deviation. A p-value of <0.05 was considered statistically significant. Statistical significance for the Mann–Whitney U test with Bonferroni’s method for the Kruskal–Wallis test was set at p<0.017 (0.05/3) for all three groups. Statistical significance for the Mann–Whitney U test with Bonferroni’s method for the Kruskal–Wallis test was divided by three, since the number of comparing the groups were three ways (mCON-mNQ, mCON-mIQ, and mNQ-mIQ).

## RESULTS

### Association of subclinical mastitis with data from automatic health monitoring and automatic milking systems

**Figure 1:**
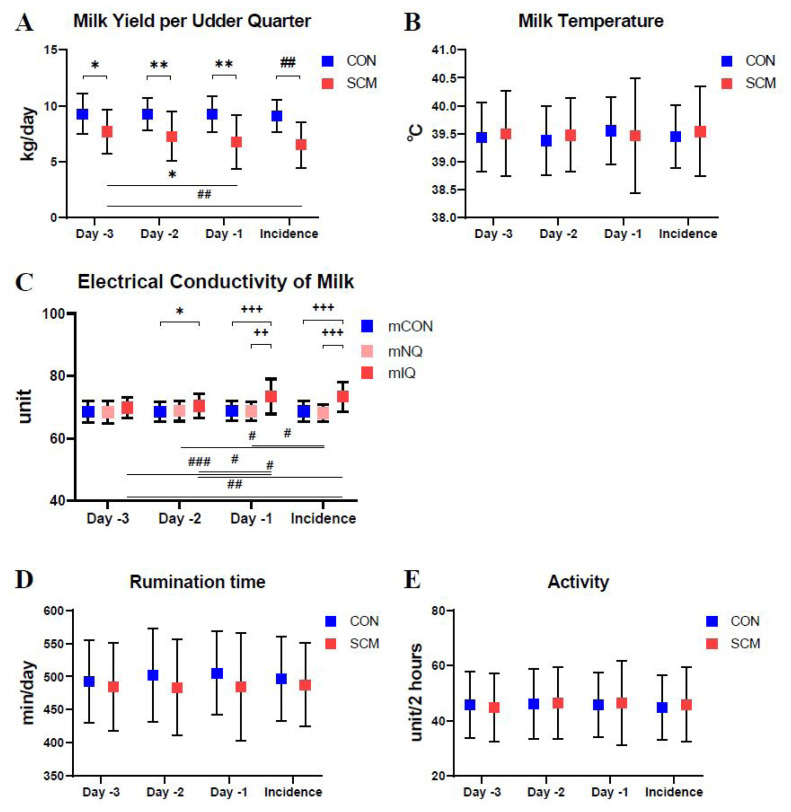
Rumination time, activity, milk yield, milk temperature, and milk conductivity in each group. CON, control group; SCM, subclinical mastitis group; mCON, milk from control group; mNQ, milk from non-inflamed quarter of the subclinical mastitis group; mIQ, milk from subclinically inflamed quarter of the subclinical mastitis group. * p<0.05; ** p<0.01 (independent t-test and paired t-test); ^##^ p<0.01 (Mann–Whitney U test with Bonferroni’s method and Wilcoxon signed rank test); ^++^ p<0.003; ^+++^ p<0.0003 (Mann–Whitney U test with Bonferroni’s method for the Kruskal–Wallis test.

The CON group had age (5.2±1.6 years), parity (2.5±1.0), days in milk (146.7±89.5 days), and body temperature (38.9°C± 0.4°C) similar to those in the SCM group (5.3±2.0 years, 2.4±1.5, 176.2±112.1 days, and 39.0°C±0.4°C, respectively) (Table 1). Daily milking frequency and body weight did not differ between the CON and SCM groups. The rumination time, activity, and milk temperature in the CON group did not differ from those in the SCM group (Figure 1). Regarding milk yield, the CON group maintained higher milk production than did the SCM group (p<0.05). In the SCM group, milk yield per udder quarter decreased on day −1 (6.76±2.44 kg/d) and the day of incidence (6.50±2.06 kg/d) compared to that on day −3 (7.70±1.97 kg/d) (p<0.05). Regarding EC, the mIQ group showed a higher EC (70.5±3.9 unit) than did the mCON group (68.5±3.3 unit) 2 days prior to the incidence (p<0.05). The mNQ group had decreased EC from day −2 (68.8±3.3 unit) to the day of incidence (68.1±2.8 unit), whereas the mIQ group had increased EC from day −3 (69.8 ±3.2 unit) to the day of incidence (73.4±4.7 unit). On day −1 and the day of incidence, the mIQ group showed differences in EC compared to the mCON and mNQ groups (p<0.003).

### Association of subclinical mastitis with hematological and serum biochemical parameters

**Figure 2:**
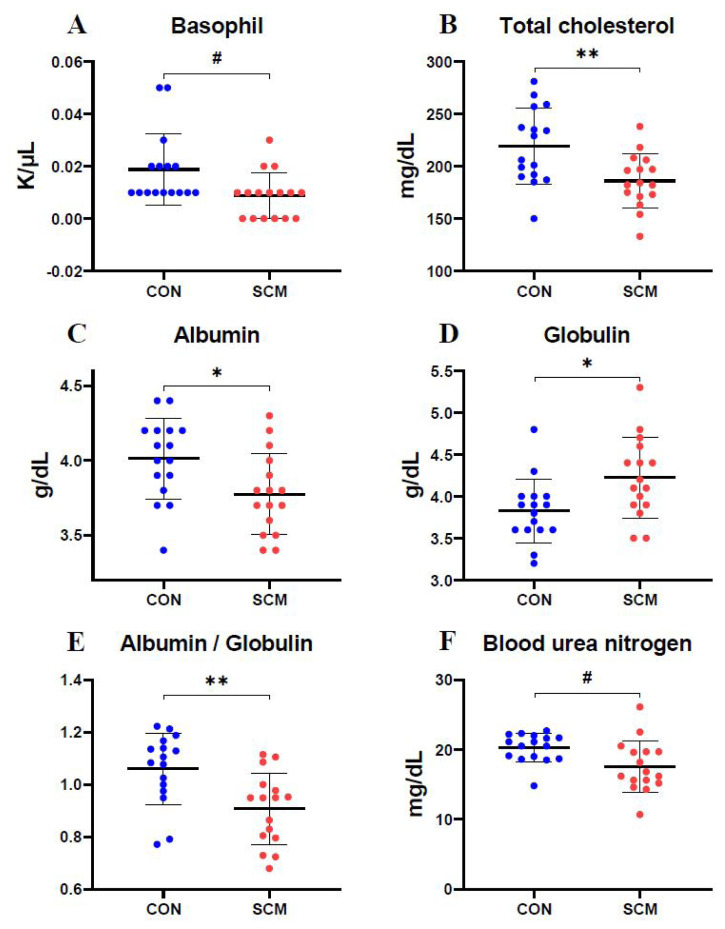
Significant results of hematological and serum biochemical parameters in each group. CON, control group; SCM, subclinical mastitis group. * p<0.05; ** p<0.01 (independent t-test); ^#^ p<0.05 (Mann–Whitney U test with Bonferroni’s method).

The SCM group differed from the CON group in terms of hematological and serum biochemical parameters (Figure 2; Supplementary Figure S1 and S2). The SCM group had lower basophil count (0.009±0.009 K/μL); total cholesterol (186.1± 25.6 mg/dL), albumin (3.78± 0.27 g/dL), and BUN (17.6±3.7 mg/dL) levels; and A/G ratio (0.91±0.14) than did the CON group (0.019±0.014 K/μL, 219.4±36.1 mg/dL, 4.01±0.27 g/dL, 20.3±2.1 mg/dL, and 1.06±0.14, respectively) (p<0.05). However, the SCM group had a higher level of globulin (4.23±0.48 g/dL) than did the CON group (3.83± 0.38 g/dL) (p<0.05).

### Association of subclinical mastitis with milk composition

**Figure 3:**
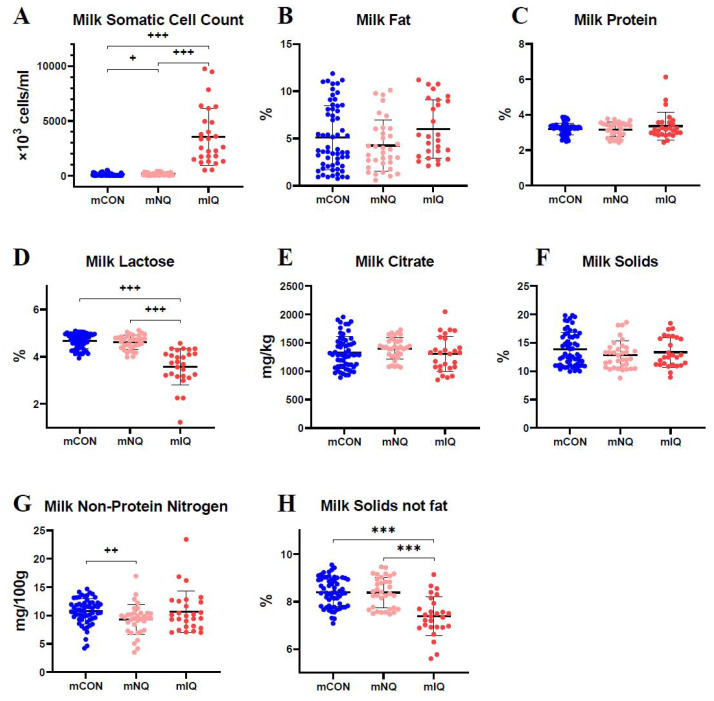
Results of milk composition analysis in each group. mCON, milk from the control group; mNQ, milk from non-inflamed quarter of the subclinical mastitis group; mIQ, milk from inflamed quarter of the subclinical mastitis group. *** p<0.001(Bonferroni post-hoc test for one-way analysis of variance); ^+^ p<0.017; ^++^ p<0.003; ^+++^ p<0.0003 (Mann–Whitney U test with Bonferroni’s method for the Kruskal–Wallis test).

The SCC, lactose, non-protein nitrogen, and milk solids-not-fat were associated with subclinical mastitis, whereas no association was found between subclinical mastitis and milk fat, protein, citrate, or total solids (Figure 3). Milk SCC was the lowest in the mCON group (97.8±118.5×10^3^ cells/mL), followed by those in the mNQ (167.0±133.8×10^3^ cells/mL) and mIQ (3,561.0±2,612.3×10^3^ cells/mL) groups (p<0.017). The mIQ group had lower levels of lactose (3.58%±0.77%) and milk solids-not-fat (7.39%±0.83%) than did the mCON (4.66%±0.32% and 8.40%±0.61%, respectively) and mNQ (4.61%±0.30% and 8.38%±0.64%, respectively) groups (p< 0.017). In addition, the mCON group had higher levels of milk non-protein nitrogen (10.7±2.2 mg/100 g) than did the mNQ group (9.3±2.6 mg/100 g) (p<0.003).

### Bacteriological findings from the udder quarter affected by subclinical mastitis

Mastitis pathogens were isolated from the mIQ group (Table 2) and no bacteria were detected in eight samples. In the remaining samples, microorganisms that normally inhabit the udders and are derived from the environment in which the cows live were isolated. The most common finding (n = 9, 33.3%) was coagulase-negative *Staphylococci*, followed by *Corynebacterium* spp. (n = 3, 11.1%), *Streptococcus* spp. (n = 3, 11.1%), and *Psychrobacter pasteurii* (n = 2, 7.4%). Among coagulase-negative *Staphylococci*, *S. simulans* (n = 4, 14.8%) was the most common pathogen, followed by *S. epidermidis* (n = 2, 7.4%) and *S. xylosus* (n = 2, 7.4%).

## DISCUSSION

In the present study, we demonstrated the characteristics of automated sensing data, hematological and serum biochemical parameters, and milk composition detected using AMS, the CMT, and milk composition analyses in dairy cows with subclinical mastitis. Subclinical mastitis was significantly associated with milk yield, EC, basophil count, A/G ratio, and levels of total cholesterol, albumin, globulin, BUN, milk SCC, milk lactose, milk non-protein nitrogen, and milk solids-not-fat. Furthermore, opportunistic or environmental mastitis pathogens were isolated from milk samples collected from the udder quarters of cows with subclinical mastitis.

Methods to identify cows with subclinical mastitis and categorize their behaviors have been developed using AMS and automatic health monitoring systems. AMS indicate subclinical mastitis by detecting decreased milk yield and abnormal EC from quarters or repeated measures between milkings [21–23]. In the present study, we identified decreased milk yield in the SCM group, increased EC in the mIQ group compared to that in the mCON group, and reduced EC in the mNQ group prior to the incidence of subclinical mastitis. An interesting finding of this study is that milk had a lower EC on the day of incidence of subclinical mastitis than a few days before the incidence in mNQ. The increased EC produced in inflamed quarters can be attributed to an increase in Na^+^ and Cl^−^ ions in milk owing to the leaky tight junctions in mammary epithelial cells [24]. Decreased concentrations of Na^+^ and Cl^−^ ions in the blood due to the leakage of Na^+^ and Cl^−^ ions in inflamed quarters may result in reduced EC in milk from mNQ. Further studies are required to elucidate why mNQ produce milk with decreased EC compared to that in the milk usually produced. In terms of automatic health-monitoring systems, a previous study reported that cows with subclinical mastitis spend less time ruminating [25]. However, in the present study, the rumination time between the CON and SCM groups did not differ, possibly because of the devices used. The device used in the present study recorded the rumination time by detecting sounds on the neck, whereas the device used in the previous study was based on the pressure of the jaw movements of cows [26,27].

Previous studies have reported increased white blood cell counts, particularly neutrophil counts, in dairy cows with subclinical mastitis [11,28,29]. However, no differences were observed in white blood cell and neutrophil counts between the CON and SCM groups in this study. The difference may be due to different experimental methods used in the studies. Other studies have used the CMT to classify cows with subclinical mastitis. However, in this study, we first detected subclinical mastitis using AMS and then determined the disease incidence using the CMT and SCC via milk composition analysis. The use of AMS and the CMT may help identify cows at an earlier stage of the disease than does the use of the CMT alone. This may have influenced the basophil counts in this study. The basophil count in the SCM group was lower than that in the CON group, although it was within the reference range and the difference was small. Decreased basophil counts are not considered clinically significant because cattle have very low basophil counts [30]. However, basophils are also involved in mastitis. During intramammary inoculation with *Escherichia coli*, dairy cows with lower circulating basophil counts reportedly developed severe mastitis [31]. Basophils might also be involved in subclinical mastitis. Further studies are required to elucidate the association between basophils and subclinical mastitis when detected by AMS.

Holstein cows respond systemically to subclinical mastitis, reflecting specific blood biochemical parameters related to inflammation and liver functions [32,33]. The inflammatory status may have contributed to the albumin and globulin levels and A/G ratio observed in this study. A decreased A/G ratio is observed when an impaired blood–milk barrier causes an increase in serum albumin levels in the milk, and globulin production is elevated in response to inflammation in cows with subclinical mastitis [33,34]. Moreover, decreased hepatic function can also influence the albumin concentration and A/G ratio, leading to decreased levels [35,36]. In addition, the total cholesterol and BUN levels indicate that subclinical mastitis may be associated with decreased hepatic functions. Cholesterol and BUN are mainly produced by the liver; therefore, decreased hepatic function results in decreased synthesis of cholesterol and BUN [35,36]. However, hepatic damage may not be associated with subclinical mastitis because hepatocellular leakage enzymes, including alanine aminotransferase, aspartate aminotransferase, and lactate dehydrogenase, which are sensitive indicators of hepatic damage, did not differ between the CON and SCM groups in this study [35].

The associations of subclinical mastitis with milk fat, protein, and total solids in previous studies [10,14,15,17] were not significant in the present study. As noted in previous studies, levels of milk lactose and solids-not-fat were lower in the mIQ group than in the mCON and mNQ groups [37,38]. Considering the differences in milk lactose and solids-not-fat between the groups, decreased lactose biosynthesis due to tissue damage in secretory cells may result in decreased levels of milk solids-not-fat [2,38]. Milk non-protein nitrogen is transferred from the blood to milk and consists of milk urea (about 50%), free amino acids, creatine, uric acid, peptides, organic acids, and phospholipids [39,40]. Interestingly, in our study, although the CON group had higher BUN levels than did the SCM group and the mCON group had higher milk non-protein nitrogen levels than did the mNQ group, no difference was observed between the mCON and mIQ groups. These findings indicated that the degree of inflammation may influence the transfer of urea nitrogen from the blood to milk.

Milk production in dairy cows can be influenced by various factors such as genetic potential, physiological status, environment, and management systems. We attempted to reduce the differences between the CON and SCM groups. We selected CON cows that had characteristics such as age, parity, and days in milk similar to those of Holstein cows raised in the same barn using the same AMS at the same time when subclinical mastitis was detected. Although milk production in the SCM group decreased due to subclinical mastitis, milk production in this group was lower than that in the CON group, regardless of the presence of the disease. A limitation of our study is that some factors that may influence milk production might not have been estimated. Future studies designed to overcome the limitations of the present study are required.

The present study was based on AMS using EC and milk production, which can help early detection of subclinical mastitis. Comparison of automated sensing data, hematological and serum biochemical parameters, and milk components between CON and SCM may provide insights into understanding the early stage of subclinical mastitis.

In conclusion, the present study demonstrated differences in subclinical mastitis using AMS. Milk yield and EC, as measured using AMS, changed before subclinical mastitis detection using SCC. On the day of disease incidence, Holstein cows with subclinical mastitis had reduced basophil count, A/G ratio, and levels of total cholesterol, albumin, and BUN and elevated globulin levels. Moreover, quarters with subclinical mastitis produced milk with elevated milk SCC and reduced milk lactose and solids-not-fat. AMS aid in the early detection of subclinical mastitis. Moreover, comparisons of automatic sensing data, hematological and serum biochemical parameters, and milk compositions between the CON and SCM groups may provide insights into the early stages of subclinical mastitis. However, further studies are required to elucidate how these variables change according to the subclinical mastitis stage and severity.

## Figures and Tables

**Table 1:** Descriptive statistics for Holstein cows according to each group in the study

Variable	CON^1)^	SCM^1)^	p-value
Number	16	16	
Age, year	5.2±1.6	5.3±2.0	0.871
Parity	2.5±1.0	2.4±1.5	0.780
Days in milk	146.7±89.5	176.2±112.1	0.417
Body temperature (°C)	38.9±0.4	39.0±0.4	0.696
Milk yield (kg/d)			
Day -3	36.5±7.4	30.8±7.9	0.043
Day -2	36.2±5.7	29.0±8.8	0.010
Day -1	36.4±6.7	27.0±9.7	0.003
Day of incidence	35.8±5.9	26.0±8.2	<0.001
Daily milking frequency			
Day -3	2.56±0.63	2.50±0.52	0.669
Day -2	2.44±0.89	2.31±0.70	0.642
Day -1	2.50±0.63	2.00±0.73	0.073
Day of incidence	2.56±0.73	2.44±0.51	0.590
Body weight			
Day -3	747.8±69.6	739.3±108.0	0.797
Day -2	738.1±70.1	741.1±102.8	0.927
Day -1	746.9±65.5	736.8±109.5	0.758
Day of incidence	746.3±65.0	739.9±113.3	0.846

Data are expressed as mean±standard deviation.

1) CON, control group; SCM, subclinical mastitis group.

**Table 2:** Bacteriological findings in milk samples from influenced udders in the subclinical mastitis group

Variable	Number	Percentage
Total	27	100
Coagulase-negative *Staphylococci*		
*Staphylococcus chromogenes*	1	3.7
*Staphylococcus epidermidis*	2	7.4
*Staphylococcus simulans*	4	14.8
*Staphylococcus xylosus*	2	7.4
*Bacillus pumilus*	1	3.7
*Corynebacterium* spp.	3	11.1
*Micrococcus luteus*	1	3.7
*Psychrobacter pasteurii*	2	7.4
*Streptococcus gallolyticus*	1	3.7
*Streptococcus uberis*	2	7.4
No growth	8	29.6

## Data Availability

The dataset generated and/or analyzed during the current study is available from the authors upon reasonable request.

## SUPPLEMENTARY MATERIAL

**Supplementary Figure S1**. Complete blood count results of each group.

**Supplementary Figure S2**. Serum biochemical parameters in each group.

## References

[1] Hogeveen H, Huijps K, Lam TJGM (2011). Economic aspects of mastitis: new developments. NZ Vet J.

[2] Adkins PRF, Middleton JR (2018). Methods for diagnosing mastitis. Vet Clin North Am Food Anim Pract.

[3] Gruet P, Maincent P, Berthelot X, Kaltsatos V (2001). Bovine mastitis and intramammary drug delivery: review and perspectives. Adv Drug Deliv Rev.

[4] Argaw A (2016). Review on epidemiology of clinical and subclinical mastitis on dairy cows. Food Sci Qual Manag.

[5] Galfi AL, Radinović MŽ, Davidov IN, Erdeljan MM, Kovačević ZR (2017). Detection of subclinical mastitis in dairy cows using California and Draminski mastitis test. Biotechnol Anim Husb.

[6] Chakraborty S, Dhama K, Tiwari R (2019). Technological interventions and advances in the diagnosis of intramammary infections in animals with emphasis on bovine population-a review. Vet Q.

[7] Neculai-Valeanu AS, Ariton AM (2022). Udder health monitoring for prevention of bovine mastitis and improvement of milk quality. Bioengineering.

[8] Malašauskienė D, Juozaitienė V, Televičius M (2019). Changes in the inline lactate dehydrogenase according to the cow’s production and reproduction status. Acta Vet Brno.

[9] Deng Z, Hogeveen H, Lam TJGM, Van der Tol R, Koop G (2020). Performance of online somatic cell count estimation in automatic milking systems. Front Vet Sci.

[10] Qayyum A, Khan JA, Hussain R, Avais M, Ahmad N, Khan MS (2016). Investigation of milk and blood serum biochemical profile as an indicator of sub-clinical mastitis in Cholistani cattle. Pak Vet J.

[11] Sadek K, Saleh E, Ayoub M (2017). Selective, reliable blood and milk bio-markers for diagnosing clinical and subclinical bovine mastitis. Trop Anim Health Prod.

[12] Siddiqe MZF, Islam S, Islam SS, Islam MS, Islam MS, Das BC (2015). Haematobiochemical changes in subclinical mastitis affected high yielding dairy cows in chittagong district. Int J Nat Soc Sci.

[13] Das D, Panda SK, Kundu AK, Jena B, Das BC, Sahu RJ (2018). Haematological and metabolic profile test of mastitis affected bovines in Odisha. J Entomol Zool Stud.

[14] Ebrahimie E, Ebrahimi F, Ebrahimi M, Tomlinson S, Petrovski KR (2018). A large-scale study of indicators of sub-clinical mastitis in dairy cattle by attribute weighting analysis of milk composition features: highlighting the predictive power of lactose and electrical conductivity. J Dairy Res.

[15] Bagri D, Pandey R, Bagri G, Kumari R, Bagdi D (2018). Effect of subclinical mastitis on milk composition in lactating cows. J Entomol Zool Stud.

[16] Batavani R, Asri S, Naebzadeh H (2007). The effect of subclinical mastitis on milk composition in dairy cows. Iran J Vet Res.

[17] Singh M, Yadav P, Sharma A, Garg VK, Mittal D (2017). Estimation of mineral and trace element profile in bubaline milk affected with subclinical mastitis. Biol Trace Elem Res.

[18] Casura C, Schukken YH, Rüsch P (1995). Quality assessment of California Mastitis Test as a diagnostic tool in quarter somatic cell count estimation.

[19] Ha S, Kang S, Jung M, Kim SB, Lee HG, Park HT (2023). Comparison of blood parameters according to fecal detection of Mycobacterium avium subspecies paratuberculosis in subclinically infected holstein cattle. J Vet Sci.

[20] Kim HJ, Youn HY, Kang HJ (2022). Prevalence and virulence characteristics of Enterococcus faecalis and Enterococcus faecium in bovine mastitis milk compared to bovine normal raw milk in South Korea. Animals.

[21] Penry JF (2018). Mastitis control in automatic milking systems. Vet Clin North Am Food Anim Pract.

[22] Khatun M, Thomson PC, Clark CEF, García SC (2020). Prediction of quarter level subclinical mastitis by combining in-line and on-animal sensor data. Anim Prod Sci.

[23] Hovinen M, Aisla AM, Pyörälä S (2006). Accuracy and reliability of mastitis detection with electrical conductivity and milk colour measurement in automatic milking. Acta Agric Scand A Anim Sci.

[24] Stelwagen K, Singh K (2014). The role of tight junctions in mammary gland function. J Mammary Gland Biol Neoplasia.

[25] Antanaitis R, Juozaitienė V, Malašauskienė D (2022). Identification of changes in rumination behavior registered with an online sensor system in cows with subclinical mastitis. Vet Sci.

[26] Gregorini P, Dela Rue BD, Pourau M, Glassey C, Jago J (2013). A note on rumination behavior of dairy cows under intensive grazing systems. Livest Sci.

[27] Norbu N, Alvarez-Hess PS, Leury BJ (2021). Assessment of rumiwatch noseband sensors for the quantification of ingestive behaviors of dairy cows at grazing or fed in stalls. Anim Feed Sci Technol.

[28] Rathaur A, Prakash V, Gupta PK, Singh SJ, Bhateshwar V (2020). Effect of subclinical mastitis in compositional change in milk and blood parameter of crossbred dairy cow. Int J Chem Stud.

[29] Saleh N, Allam TS, Omran A, Abdelfattah AM (2022). Evaluation of changes in hemato-biochemical, inflammatory, and oxidative stress indices as reliable diagnostic biomarkers for subclinical mastitis in cows. Alex J Vet Sci.

[30] Stockham SL, Scott MA (2013). Fundamentals of veterinary clinical pathology.

[31] Pezeshki A, Stordeur P, Wallemacq H (2011). Variation of inflammatory dynamics and mediators in primiparous cows after intramammary challenge with Escherichia coli. Vet Res.

[32] Pegolo S, Giannuzzi D, Piccioli-Cappelli F (2023). Blood biochemical changes upon subclinical intramammary infection and inflammation in Holstein cattle. J Dairy Sci.

[33] Matei ST, Groza I, Andrei S, Bogdan L, Ciupe S, Petrean A (2010). Serum metabolic parameters in healthy and subclinical mastitis cows. Bull Univ Agric Sci Vet Med Cluj Napoca.

[34] Wellnitz O, Bruckmaier RM (2021). Invited review: the role of the blood–milk barrier and its manipulation for the efficacy of the mammary immune response and milk production. J Dairy Sci.

[35] Latimer KS (2011). Duncan and Prasse's veterinary laboratory medicine: clinical pathology.

[36] Bertoni G, Trevisi E (2013). Use of the liver activity index and other metabolic variables in the assessment of metabolic health in dairy herds. Vet Clin North Am Food Anim Pract.

[37] Tomazi T, Gonçalves JL, Barreiro JR, Arcari MA, Dos Santos MV (2015). Bovine subclinical intramammary infection caused by coagulase-negative staphylococci increases somatic cell count but has no effect on milk yield or composition. J Dairy Sci.

[38] Gonçalves JL, Tomazi T, Barreiro JR (2016). Effects of bovine subclinical mastitis caused by Corynebacterium spp. on somatic cell count, milk yield and composition by comparing contralateral quarters. Vet J.

[39] Gasqui P, Trommenschlager JM (2017). A new standard model for milk yield in dairy cows based on udder physiology at the milking-session level. Sci Rep.

[40] Ruska D, Jonkus D (2014). Crude protein and non-protein nitrogen content in dairy cow milk. Proc Latv Univ Agric.

