# Supplementary material for: Effects of subclinical mastitis on automatic milking system data, hematological and biochemical parameters, and milk composition in Holstein cows
Source: Anim Biosci. 2024 Aug 27;38(1):166–75. doi: 10.5713/ab.24.0460 (PMC11725738; doi:10.5713/ab.24.0460)
Supplement: Supplementary file 2 [file ab-24-0460-Supplementary-Fig-2.pdf]

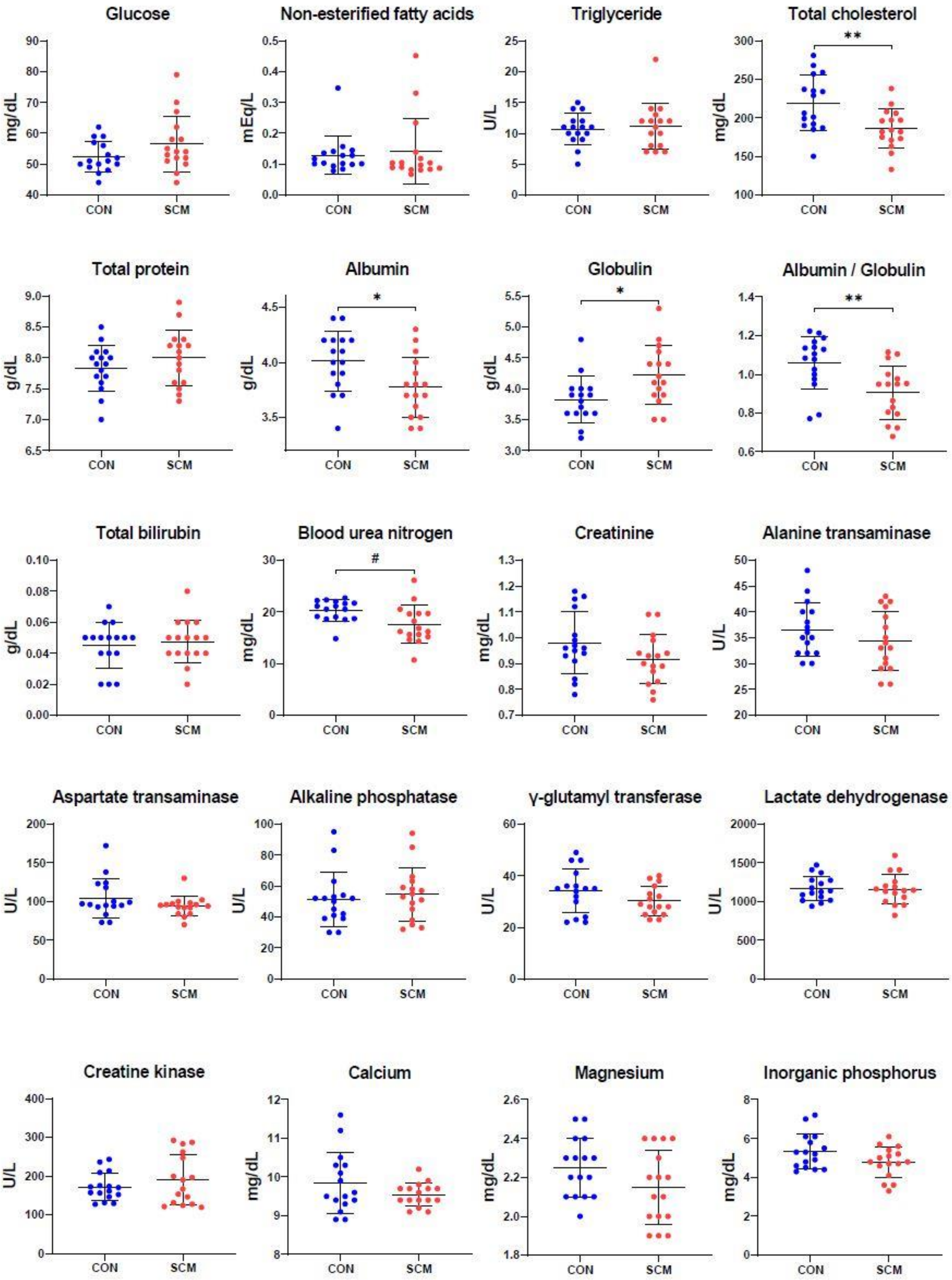

Supplementary Figure 2. Serum biochemical parameters in each group

CON, control group; SCM, subclinical mastitis group

*p<0.05; **p<0.01 (independent t-test)

#p<0.05 (Mann–Whitney U test with Bonferroni's method)
